# Factors driving public tolerance levels and information‐seeking behaviour concerning insects in the household environment

**DOI:** 10.1002/ps.4839

**Published:** 2018-02-23

**Authors:** Bruce Schoelitsz, P Marijn Poortvliet, Willem Takken

**Affiliations:** ^1^ Kennis‐ en Adviescentrum Dierplagen Wageningen The Netherlands; ^2^ Laboratory of Entomology Wageningen University and Research Wageningen The Netherlands; ^3^ Strategic Communication Wageningen University and Research Wageningen The Netherlands

**Keywords:** integrated pest management, risk perception, information‐seeking behaviour, household insects, humans, risk information seeking and processing

## Abstract

**BACKGROUND:**

The public's negative attitudes towards household insects drive tolerance for these insects and their control. Tolerance levels are important in integrated pest management (IPM), as are pest knowledge and information. The risk information seeking and processing (RISP) model describes the relationships between personal factors and information‐seeking behaviour. We combined IPM and RISP to determine important relationships between factors driving insect tolerance levels and information‐seeking behaviour through an online survey and tested whether this model is valid and generally applicable.

**RESULTS:**

Relationships between variables from both IPM and RISP models were tested for seven insect species. Tolerance levels were measured with two factors: willingness to pay for pest control and whether insects are tolerated. Willingness to pay for control was positively affected by age, experience, risk perception, insect characteristics, and negative emotions and affected behavioural intention, by influencing information sufficiency and information‐seeking behaviour. Tolerability was influenced by perception of insect characteristics and determines whether control measures are taken.

**CONCLUSION:**

It was possible to combine the RISP and IPM models. Relevant driving factors were a person's age, experience, risk perception, negative affective responses, tolerance levels, relevant channel beliefs about online forums, information sufficiency and information‐seeking behaviour. There was, however, variation in important factors between different insects. © 2017 The Authors. *Pest Management Science* published by John Wiley & Sons Ltd on behalf of Society of Chemical Industry.

## 1 INTRODUCTION

Insects in the household environment may pose a risk to humans by vectoring parasitic or pathogenic agents as well as damaging physical constructions, materials and food.^1^, ^2^, ^3^ Furthermore, insects are considered a nuisance because of their abundance as well as for aesthetic or emotional reasons.^4^, ^5^, ^6^ Preventing, reducing or assessing the risks from insects in the household environment requires knowledge and expertise that the public usually does not possess. Attitudes of the public towards insects and other arthropods are mainly negative and may interfere with a fact‐based assessment of the risks posed. Feelings of fear, dislike and aversion are often expressed.^7^, ^8^ These negative attitudes are reflected in low tolerance levels (TLs) within the domestic environment, which are often (close to) zero.^9^ For example, even low numbers of the oriental cockroach (*Blatta orientalis* L.) within apartments are regarded as a problem by many and a maximum of none to only one cockroach is tolerated indoors.^10^, ^11^, ^12^ Similarly, people have a strong aversion to bed bugs (*Cimex lectularius* L.), and will go to great lengths to get rid of them. Personal factors, such as the feelings described above, affect a person's information seeking or pest‐management behaviour. The relationship between various personal factors, a person's TL for household insects and the resulting behaviour has not yet been studied using an integrative approach.

Perception of and attitude towards arthropods (i.e. insects, spiders, mites and ticks) affect insect tolerance and control behaviour. The low TL for insects in homes is reflected in the high percentage of the US and European population that use pesticides, mainly insecticides, within the household environment.^11^, ^13^, ^14^, ^15^, ^16^, ^17^ The use of insecticides, however, poses health risks, especially for young children.^18^ Moreover, the product used can remain in the environment for extended periods of time,^17^ it may not be the most effective method, especially when used on its own,^19^ and insects have developed resistance to some insecticides.^20^ Therefore, understanding residents' attitudes to and knowledge of household pest species is of importance for the development of sustainable pest management programmes.^21^, ^22^

Tolerance or threshold levels are a driver for decision taking in integrated pest management (IPM). IPM is a rational, common‐sense approach to sustainable pest control. The purpose of establishing threshold levels is to define a pest population level that can be tolerated.^9^ If this level is exceeded, control measures are implemented. In addition to the establishment of threshold levels, the National Pest Management Association (NPMA) identifies four actions within the IPM of urban pests: inspection, identification, and, when the threshold is exceeded, employment of two or more appropriate control measures and evaluation of effectiveness.^9^ It can hardly be expected that the sequence of steps within IPM also be taken by the public, but, in reality, these steps do reflect the general process of pest control by lay people. After encountering one or multiple insects (one of the goals of an inspection), they are identified (be that correct or incorrect), and when the TL is exceeded, control measures are taken. If, after these measures, the insects are still present (evaluation of effectiveness), this continuous process starts with the first action again. In order to manage pests sustainably, i.e. prevent further establishment and development of pest insects and minimise chemical control methods, knowledge and information about the pest species and possible control measures are required.

The risk information seeking and processing (RISP) model applies a bottom‐up approach to studying the relationship between risk perception and information‐seeking behaviour (ISB).^23^ The model considers demographics, risk perception, affective responses (ARs) to potential risks, and subjective norms. It then tests how these factors affect each other as well as individuals' needs for information, their ability to find this information and their evaluation of the information sources. The last three factors influence ISB. ISB may eventually affect the behavioural intention (BI), for which the RISP model is a precursor.^23^, ^24^ The model has been used to study the relationship between risk perception and information seeking in a variety of areas, including changing from a Chinese to an American‐style diet,^25^ health care,^24^ and climate change.^26^

We used the RISP model to study what factors influence TLs concerning household insects, and whether this tolerance affects information‐seeking and management behaviour. The processes as described in the RISP model are initiated directly after the identification of one or multiple encountered insects.^23^

The RISP model can broadly be divided into two parts, i.e. personal factors that influence the need for information and the factors that influence ISB. The logical position for the TL, being affected by subjective personal factors and functioning as an action threshold, would be in between these two parts of the RISP model. The RISP model is an antecedent of BI. In this study, BI is composed of both preventive and curative (extermination or removal of insects) control measures, i.e. an action in the process of IPM.

**Figure 1:**
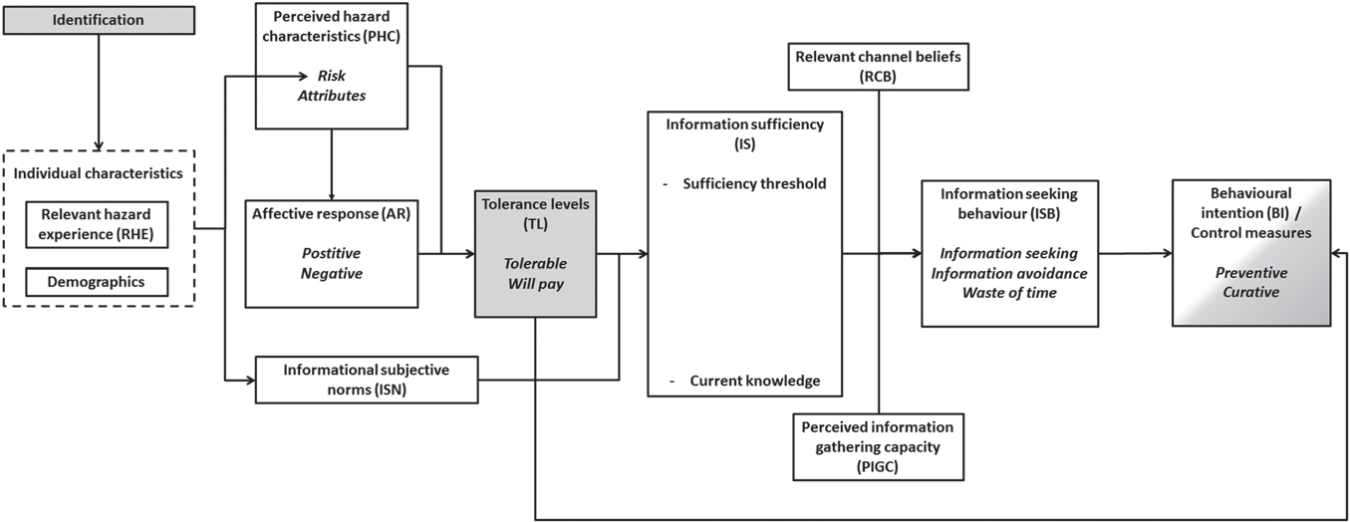
The integrated model in which variables from the RISP model (white) and IPM model (grey) are shown.

The integrated model is shown in Figure 1 (see Table 1 for an overview of definitions and abbreviations of the variables). Individual characteristics [relevant hazard characteristics (RHC) and demographics] affect both perceived hazard characteristics (PHC) and informational subjective norms (ISN). PHC influences the AR, but instead of these emotions affecting information sufficiency (IS), we hypothesised that AR and PHC together affect TL (adapted from the threshold level, a parameter from the IPM framework) of the encountered insect species. TL and ISN influence IS, i.e. the amount of extra information needed to be able to deal with the risk. Together with the relevant channel beliefs (RCB) and perceived information‐gathering capacity (PIGC), IS affects ISB, which in turn drives BI, which is a proximal predictor of behaviour.^27^

**Table 1:** Definitions of abbreviations and parameters of the integrated model, after Griffin^23^ and Sarisky^9^

RISP	Risk information seeking and processing 23
IPM	Integrated pest management 9
Individual characteristics	Attributes of a person that affect that individual's view of the characteristics of a given hazard 23
Relevant hazard experience (RHE)	A person's past experience with the same hazard or with what a person might nominate as a related hazard 23
Perceived hazard characteristics (PHC)	A person's view of the characteristics of a given hazard or risk 23
Affective response (AR)	A person's response that could influence the amount of information needed to take effective action 23
Informational subjective norms (ISN)	The perceived social influences motivating the desire for information sufficiency 23
Tolerance level (TL)	Site‐specific insect population size that is tolerated 9
Information sufficiency (IS)	The difference between the amount of information a person already possesses and the amount of information that is thought to be needed to adequately manage the risk 23
Relevant channel beliefs (RCB)	A person's opinion on the trustworthiness and usefulness of information sources 23
Perceived information gathering capacity (PIGC)	A person's expected ability to perform the information‐seeking behaviour 23
Information seeking behaviour (ISB)	Attempts to gather or avoid risk information 23
Behavioural intention (BI)	Predictor for actual performance of insect management behaviour 23

In the present study, we integrated factors of the RISP model within IPM and collected data from an online survey carried out among the public in The Netherlands (1) to test whether the proposed model is valid, (2) to explore what driving factors are most important, and (3) to determine whether the model can be generalised or is insect specific.

## 2 EXPERIMENTAL METHODS

### 2.1 Sampling and participants

An online survey was developed with qualtrics survey software (Qualtrics, Provo, UT, USA). A short article about the presence of insects commonly found within the household environment, containing an obvious link to the survey, was provided to 98 local newspapers throughout The Netherlands. Positive feedback was received from 24 newspapers, indicating their willingness to publish the article in print or online. At least six more newspapers published the article without providing feedback.

A total of 382 persons started the survey, of whom 263 respondents (68.8%) completed all the questions. The survey sample consisted of a higher percentage of women, a higher percentage of those with a higher education degree, and a higher percentage of home‐owners than the general Dutch population. The percentage of respondents living with children (< 18 years old) was lower than in the Dutch population. Nearly half of the respondents kept pets and only a small minority had involvement with insects professionally (Table 2). The distribution of adult age resembled that of Dutch society.

**Table 2:** Descriptive statistics for respondent characteristics (CBS Statistics Netherlands; http://www.cbs.nl, visited on 10 July 2017)

Characteristic	Survey population (%)	Dutch population (%)
Gender	31.9 male/68.1 female	49.6 male/50.1 female
Educational degree ≥ Bachelor	56.6	26.8
Home owner	74.1	57.0
Living with children	25.5	53.6
Pet owner	47.1	‐
Professional involvement with insects	4.3	‐

### 2.2 Procedure

The survey consisted of 41 questions. Eight questions concerned demographics and 33 questions assessed the remaining variables of the integrated model. Respondents were asked to provide answers about seven selected insects which are found in households in The Netherlands: housefly (Musca domestica L.), grey silverfish (Ctenolepisma longicaudata Escherich), bed bug, ladybird beetle (Coccinellidae), biscuit beetle (Stegobium paniceum L.), common furniture beetle (Anobium punctatum De Geer), and cockroach (Blattodea). The selection of insects was based on characteristics that are associated with pest status and their occurrence within the household environment, as described below.

The housefly (M. domestica) is a widespread species that is capable of flying, can appear in large numbers, has the potential to spread diseases and develops outdoors as well as indoors.^28^ The grey silverfish (C. longicaudata) is a species common in modern buildings. The species can be present in large numbers and can damage materials such as books and fabrics. This species lacks wings and outdoor development has never been described in The Netherlands.^29^ The bed bug (C. lectularius) is an ectoparasite of which all mobile stages feed on vertebrate blood. Their biting behaviour may cause dermal irritation, leading to a swelling of the skin. This crawling species develops indoors and is generally thought of as being difficult to control.^6^ The biscuit beetle (S. paniceum) is a common pest of stored food. They are found in a wide selection of products, and especially the larvae are known to damage dried food and spices. Development occurs indoors,^30^ and may occur outdoors as well. Female common furniture beetles (A. punctatum) oviposit in cracks and crevices of wood. The larvae feed and develop in wooden furniture and wood used in construction. Activity of this species is generally not observed until small emergence holes appear in the wood.^3^ Of the cockroaches present in The Netherlands, the German cockroach (Blattella germanica L.) is the most common. This potential vector of food‐borne diseases develops indoors, where populations can reach large numbers.^30^ Although equipped with wings, indoor‐dwelling cockroaches found in The Netherlands do not fly. The ladybird beetle (Coccinellidae) was introduced in the survey as an antagonist to the species considered pests. Although ladybird beetles can be a nuisance because of their presence in large numbers,^31^ they are generally well tolerated and not considered a pest.^32^

### 2.3 Variables tested

The variables, except for demographics, were measured on a seven‐point Likert scale with at least two questions. Cronbach's alpha was used to test whether the different questions of a single variable could be collapsed into a single index, as it measures the internal consistency between the different items.^33^, ^34^ The questions of a variable were considered a group and pooled if values for Cronbach's alpha were ≥0.6. When values were <0.6 for the majority of insects, the different questions were regarded as separate variables. See Appendix 1 for the list of questions.

Respondents were shown a photograph of the selected insects, with a short description, and were asked whether they knew the depicted insect (yes or no).

Relevant hazard experience (RHE) was tested with two questions (Cronbach's alpha ≥0.6 for all insects).

The AR was assessed with three positive (comfortable, enthusiastic and content) and four negative (anxious, ashamed, worried and uncomfortable) feelings.^33^, ^34^ The feelings were pooled as positive (AR pos) and negative (AR neg) feelings (Cronbach's alpha ≥0.6 for all insects for both positive and negative feelings).

PHC was measured with multiple variables. Participants' risk perception (PHC risk) was measured by multiplying the values of the severity of the consequences of the selected insects being present by the perceived chance of encountering these insects within the home. Furthermore, PHC was measured by the severity of the following insect characteristics (PHC attributes): damaging food, damaging materials, damaging construction, stinging or biting, vector of disease‐causing pathogens, being plentiful, looking scary, looking disgusting, flying, crawling or jumping, indoor reproduction, outdoor reproduction, and being difficult to control.

ISN was tested with two questions (Cronbach's alpha ≥0.6 for all insects).

TL was tested with two questions that could not be pooled (Cronbach's alpha <0.6 for all insects). Both aspects were considered variables: ‘To what extent do you consider the presence of these insects in your home tolerable?’ (TL tolerable) and ‘To what extent are you willing to pay to get rid of these insects at home?’ (TL will pay).

To measure the IS, the current knowledge (measured with two questions; Cronbach's alpha ≥0.6 for all insects) was subtracted from the sufficiency threshold (measured with two questions; Cronbach's alpha ≥0.6 for all insects).

PIGC was measured with two questions (Cronbach's alpha ≥0.6).

The RCB of 12 sources of information was measured with four questions (Cronbach's alpha ≥0.6 for all sources). The sources of information consisted of family, neighbours, pest control operators (PCOs), employees of local government, employees of scientific institutions (universities, museums and centres of expertise), insecticide labels or salespersons, books, newspapers or television, PCO websites, local government websites, scientific institution websites, and online forums.

ISB was divided into information seeking and information avoidance. Both were measured with two questions that could be combined for information seeking (Cronbach's alpha ≥0.6), but not for information avoidance (Cronbach's alpha <0.6), which was split into ‘avoiding information about these insects’ (IA avoidance) and ‘seeking information about these insects is a waste of time’ (IA waste of time).

BI was measured with two variables, both consisting of two questions: preventive behaviour (BI preventive) and curative behaviour (BI curative).

### 2.4 Statistical analyses

To model the relationship between the different variables based on the proposed integrative model, ordinary least square regression analyses with multiple predictors were performed for each insect (Table 3).

**Table 3:** Dependent variables and independent variables for the different steps of the proposed model

	Dependent variable	Predictor variables
1	Perceived hazard characteristics (risk)	Relevant hazard experience, demographics
2	Informative subjective norms	Relevant hazard experience, demographics
3a	Positive affective response	Perceived hazard characteristics (attributes), perceived hazard characteristics (risk)
3b	Negative affective response	Perceived hazard characteristics (attributes), perceived hazard characteristics (risk)
4a	Tolerance level (tolerable)	Perceived hazard characteristics (attributes), perceived hazard characteristics (risk), positive affective response, negative affective response
4b	Tolerance level (will pay)	Perceived hazard characteristics (attributes), perceived hazard characteristics (risk), positive affective response, negative affective response
5	Information sufficiency	Tolerance level (tolerable), tolerance level (will pay), informative subjective norms
6a	Information‐seeking behaviour	Perceived information gathering capacity, relevant channel beliefs, information sufficiency
6b	Information avoidance (waste of time)	Perceived information gathering capacity, relevant channel beliefs, information sufficiency
6c	Information avoidance (avoidance)	Perceived information gathering capacity, relevant channel beliefs, information sufficiency
7a	Behavioural intention (preventive)	Information‐seeking behaviour, information avoidance (waste of time), information avoidance (avoidance)
7b	Behavioural intention (curative)	Information‐seeking behaviour, information avoidance (waste of time), information avoidance (avoidance)
8a	Behavioural intention (preventive)	Tolerance level (tolerable), tolerance level (will pay)
8b	Behavioural intention (curative)	Tolerance level (tolerable), tolerance level (will pay)

Perceived hazard characteristics (risk) is calculated as the product of the value for severity of the insect's presence and the chance of encountering selected insects in the home. Perceived hazard characteristics (attributes) is the perceived severity of different insect characteristics. Tolerance level (tolerable) is measured as the extent to which insect presence is tolerated. Tolerance level (will pay) is measured as the extent to which a person is willing to pay for control measures. Information avoidance (waste of time) is measured as the extent to which seeking information about the subject is a waste of time. Information avoidance (avoidance) is measured as the extent to which information about the subject is avoided. Behavioural intention (curative) is measured as the extent to which a person will exterminate or remove household insects, and behavioural intention (preventive) is measured as the extent to which a person takes measures to prevent nuisance from these insects.

To test for differences between insects, insect characteristics or sources of information, a Levene's test of homogeneity of variances was performed. If variances were equal across groups (P < 0.05), differences between groups were analysed with the analysis of variance (ANOVA) and the Tukey post hoc test. In cases where the assumption of homogeneity was not met, data were analysed with the Welch test and a Games–Howell post hoc test. Statistical analyses were performed with imb spss Statistics for Windows, Version 20.0 (IBM Corp, Armonk, NY, USA).

## 3 RESULTS

Both the descriptive results (Tables 4 and 5) and the results of the regression analyses (Tables 6, 7, 8, 9, 10, 11, 12) are explained per dependent variable.

**Table 4:** Mean (± standard deviation) values of variables from the proposed model on a seven‐point Likert scale

		Housefly	Grey silverfish	Bed bug	Cockroach	Furniture beetle	Biscuit beetle	Ladybird beetle
Insect known (%)		98.5	86.3	38.4	79.8	69.6	15.6	99.6
RHE		4.3 ± 1.5^a^	4.0 ± 2.2^a^	1.1 ± 0.6^b^	1.2 ± 0.8^bc^	1.4 ± 1.1^c^	1.1 ± 0.5^b^	2.1 ± 1.1^d^
AR	Positive	1.4 ± 0.9^a^	1.3 ± 0.8^ab^	1.3 ± 0.8^b^	1.2 ± 0.6^bc^	1.2 ± 0.7^ab^	1.3 ± 0.9^ac^	2.5 ± 1.6^d^
	Negative	2.0 ± 1.1^a^	2.8 ± 1.5^b^	4.6 ± 1.8^c^	4.8 ± 1.7^c^	3.6 ± 1.6^d^	3.2 ± 1.8^bd^	1.3 ± 0.7^e^
PHC	Risk	18.5 ± 15.4^a^	25.2 ± 16.8^b^	28.1 ± 17.9^bc^	29.0 ± 18.5^bc^	29.8 ± 17.2^c^	18.5 ± 16.1^a^	5.2 ± 7.6^d^
ISN		2.6 ± 1.8^ab^	3.4 ± 2.0^cd^	3.4 ± 2.1^cd^	3.5 ± 2.2^c^	3.3 ± 2.1^cd^	2.9 ± 2.0^ad^	2.3 ± 1.6^b^
TL	Tolerable	3.2 ± 1.7^a^	2.3 ± 1.6^b^	1.5 ± 1.5^cd^	1.5 ± 1.4^d^	1.6 ± 1.5^cd^	1.9 ± 1.5^bc^	4.3 ± 2.0^e^
	Will pay	2.6 ± 1.9^a^	4.2 ± 2.2^b^	5.5 ± 1.9^c^	6.0 ± 1.7^c^	5.7 ± 1.8^c^	4.2 ± 2.1^b^	1.8 ± 1.5^d^
IS		−2.2 ± 3.0^a^	1.0 ± 3.0^b^	2.0 ± 3.0^c^	1.5 ± 3.2^bc^	1.8 ± 3.1^bc^	2.3 ± 2.9^c^	−0.4 ± 3.3^d^
ISB	Information seeking	2.9 ± 1.8^a^	4.9 ± 1.8^b^	6.1 ± 1.6^c^	6.1 ± 1.6^c^	6.0 ± 1.5^c^	5.5 ± 1.7^d^	3.0 ± 1.8^a^
	Information avoidance	3.4 ± 2.5^a^	2.1 ± 1.6^b^	1.7 ± 1.4^bc^	1.6 ± 1.4^c^	1.6 ± 1.3^c^	1.8 ± 1.4^bc^	3.4 ± 2.4^a^
	Waste of time	1.7 ± 1.6^ab^	1.4 ± 1.1^b^	1.4 ± 1.2^b^	1.4 ± 1.1^b^	1.4 ± 1.1^b^	1.5 ± 1.2^b^	1.9 ± 1.8^a^
BI	Preventive	4.4 ± 2.2^a^	3.7 ± 2.2^bc^	3.8 ± 2.4^bc^	4.1 ± 2.5^ab^	3.4 ± 2.4^c^	3.2 ± 2.3^c^	2.2 ± 1.9^d^
	Curative	4.5 ± 2.3^a^	5.2 ± 2.0^b^	6.3 ± 1.5^c^	6.5 ± 1.1^c^	6.4 ± 1.2^c^	5.7 ± 1.8^d^	3.6 ± 2.4^e^

Values for the number of people who know the depicted insect are presented as percentages. PHC risk is calculated by multiplying the value for severity of the insect's presence and the chance of encountering selected insects in the home. IS is calculated by subtracting the current knowledge from the sufficiency threshold. TL (tolerable) is measured as the extent to which insect presence is tolerated, TL (will pay) is measured as the extent to which a person is willing to pay for control measures, ISB (waste of time) is measured as the extent to which seeking information about the subject is a waste of time, ISB (avoidance) is measured as the extent to which information about the subject is avoided, BI (curative) is measured as the extent to which a person will exterminate or remove household insects, and BI (preventive) is measured as the extent to which a person takes measures to prevent nuisance by these insects. Significant differences between insects are indicated by different superscript letters.

RHE, relevant hazard experience; AR, affective response; PHC, perceived hazard characteristics; ISN, informational subjective norms; TL, tolerance level; IS, information sufficiency; ISB, information‐seeking behaviour; BI, behavioural intention.

**Table 5:** Mean (± standard deviation) of variables of PHC attributes concerning insects in the household environment, RCB for a variety of sources of information and PIGC on a seven‐point Likert scale

PHC	Damage materials	5.9 ± 1.6^a^	RCB	PCO websites	5.7 ± 1.2^a^
	Damage food	5.8 ± 1.6^a^		PCOs	5.1 ± 1.3^b^
	Difficult to control	5.7 ± 1.5^a^		Insecticide label or salespersons	4.7 ± 1.4^b^
	Vector of pathogenic agents	5.5 ± 1.9		Family	4.6 ± 1.4^c^
	Damage construction	5.5 ± 1.8^ab^		Scientific institution websites	4.6 ± 1.8^c^
	Stinging or biting	5.5 ± 1.8^ab^		Local government websites	4.5 ± 1.7^c^
	Indoor development	5.4 ± 1.9^ab^		Online forum	4.5 ± 1.7^c^
	They are plentiful	5.2 ± 1.8^b^		Books	4.3 ± 1.4^cd^
	They look disgusting	4.5 ± 2.1^c^		Local government employees	4.0 ± 1.5^de^
	Crawling or jumping	4.4 ± 2.2^c^		Neighbours	3.8 ± 1.6^e^
	Flying	4.0 ± 2.2^c^		Newspapers or television	3.7 ± 1.5^e^
	They look scary	4.0 ± 2.2^c^		Scientific institution employees	3.7 ± 1.5^e^
	Outdoor development	3.3 ± 2.2^d^	PIGC		5.1 ± 1.5

Significant differences between variables within items are indicated by different superscript letters. PHC, perceived hazard characteristics; RCB, relevant channel beliefs; PIGC, perceived information gathering capacity.

**Table 6:** The relationships between demographic factors and relevant hazard experience (RHE) and the dependent variables risk perception [perceived hazard characteristics (PHC) risk] and informational subjective norms (ISN) for different insects

	Predictors					
	Demographics					
Dependent	Gender	Age	Education	Children	Pets	RHE
**PHC risk**						
Housefly	0.12*	0.18**	−0.13†	0.12†		0.41***
Grey silverfish		0.11†				0.55***
Bed bug		0.15*		0.16*		0.11†
Cockroach	0.10†	0.19**	−0.32*	0.12†		
Furniture beetle						0.15*
Biscuit beetle	0.10†	0.18**	−0.14*	0.14*		
Ladybird beetle	0.10†				0.13*	0.25***
**ISN**						
Housefly		0.12*			−0.16*	0.21***
Grey silverfish		0.14*				0.31***
Bed bug		0.12†				
Cockroach						
Furniture beetle						
Biscuit beetle						
Ladybird beetle						0.25***

Significant (*P < 0.05; **P < 0.01; ***P < 0.001) and marginally significant (†P < 0.1) standardised regression coefficients (beta) are presented. Blank spaces represent non‐significant betas. PHC risk is calculated by multiplying the value for severity of the insect's presence and the chance of encountering selected insects in the home.

**Table 7:** The relationships between perceived hazard characteristics (PHC) of insect attributes (attributes) and perceived risk (risk), and the dependent variables positive affective response (AR pos) and negative affective response (AR neg) for different insects

	Predictors										
	PHC attributes										PHC
Dependent	Food	Materials	Construction	Vector	Scary	Flying	Crawling or jumping	Indoor reproduction	Outdoor reproduction	Difficult to control	Risk
**AR pos**											
Housefly	−0.21*		0.23*			−0.23*	0.22†				
Grey silverfish							0.22†	−0.16†			
Bed bug				0.23*			0.22†				
Cockroach								−0.16†	0.17*		
Furniture beetle											
Biscuit beetle	−0.20*										
Ladybird beetle		−0.23*	−0.25*			−0.25*	0.21†				
**AR neg**											
Housefly		−0.22**	0.28***								0.47***
Grey silverfish			0.22**	−0.13†			0.21*				0.57***
Bed bug					0.24**					0.19*	0.28***
Cockroach					0.19*			0.15†			0.28***
Furniture beetle					0.28***				0.13†		0.32***
Biscuit beetle				−0.12†	0.22**						0.44***
Ladybird beetle										−0.18*	0.54***

Significant (*P < 0.05; **P < 0.01; ***P < 0.001) and marginally significant (†P < 0.1) standardised regression coefficients (beta) are presented. Blank spaces represent non‐significant betas. PHC risk is calculated by multiplying the value for severity of the insect's presence and the chance of encountering selected insects in the home.

**Table 8:** The relationships between perceived hazard characteristics (PHC) of insect attributes, perceived risk, positive affective response (AR pos) and negative affective response (AR neg) and the dependent variables tolerance level (TL) measured as the extent to which the presence of insects is tolerated (tolerable) and TL measured as the extent to which a person is prepared to pay for control measures (will pay) for different insects

Dependent	Predictors												
	PHC attributes									PHC		AR	
	Food	Construction	Vector	Plentiful	Scary	Disgusting	Flying	Crawling or jumping	Indoor reproduction	Outdoor reproduction	Risk	Pos	Neg
**TL tolerable**													
Housefly		0.17†					−0.38***	0.33**			−0.13†	0.25***	
Grey silverfish					0.26**	−0.35***	−0.45***	0.49***			−0.21*		
Bed bug					0.36***	−0.44***	−0.27*	0.28*					−0.19**
Cockroach			−0.15†	0.17†	0.35***	−0.42***	−0.33**	0.27*					
Furniture beetle	0.18*		−0.19*	0.24**	0.36***	−0.46***	−0.30*	0.24†					
Biscuit beetle					0.22*	−0.40***	−0.32**	0.23†				0.18**	
Ladybird beetle					−0.14†	0.20*				−0.23***		0.42***	−0.20**
**TL will pay**													
Housefly								−0.19†			0.17*		0.31***
Grey silverfish						0.26**					0.42***		0.21***
Bed bug						0.22*		−0.23*	0.18*		0.18**		0.29***
Cockroach					−0.21*	0.41***					0.17**		0.24***
Furniture beetle		0.15†		−0.16†	−0.17†	0.32**					0.18**		0.20**
Biscuit beetle				−0.13†	−0.24**	0.43***					0.22**		0.25***
Ladybird beetle											0.19†	−0.12*	0.36***

Significant (*P < 0.05; **P < 0.01; ***P < 0.001) and marginally significant (†P < 0.1) standardised regression coefficients (beta) are presented. Blank spaces represent non‐significant betas.

**Table 9:** The relationships between tolerance level (TL) measured as the extent to which the presence of insects is tolerated (tolerable), TL measured as the extent to which a person is prepared to pay for control measures (will pay) and informational subjective norms (ISN) and the dependent variable information sufficiency (IS) for different insects

	Predictors		
	TL		
Dependent	tolerable	will pay	ISN
**IS**			
Housefly	−0.13*	0.16*	0.11†
Grey silverfish		0.39***	
Bed bug		0.21**	
Cockroach		0.18**	
Furniture beetle		0.22***	
Biscuit beetle	−0,12†	0.30***	
Ladybird beetle	−0.11†	0.23***	

Significant (*P < 0.05; **P < 0.01; ***P < 0.001) and marginally significant (†P < 0.1) standardised regression coefficients (beta) are presented. Blank spaces represent non‐significant betas.

**Table 10:** The relationships between perceived information gathering capacity (PIGC), relevant channel beliefs (RCB) for several sources and information sufficiency (IS) and the dependent variables information‐Seeking behaviour (ISB), information avoidance (IA) measured as the extent to which seeking information about the subject is a waste of time (waste of time), and IA measured as the extent to which information about the subject is avoided (avoidance) for different insects

	Predictors											
		RCB										IS
Dependent	PIGC	Family	PCO	Employees of scientific institutions	Insecticide label or salespersons	Books	Newspapers or television	PCO website	Scientific institution website	Local government website	Online forum	
**ISB**											
Housefly		0.21**	0.14†						0.24*		0.13*	0.35***
Grey silverfish								0.14†			0.21**	0.37***
Bed bug				0.19†			0.18*	0.19*			0.14*	0.22***
Cockroach								0.19**			0.13†	0.28***
Furniture beetle	0.11†							0.26**			0.16*	0.26***
Biscuit beetle	0.11†				0.13*		0.11†				0.11†	0.32***
Ladybird beetle									0.19†		0.14*	0.22***
**IA waste of time**												
Housefly	−0.18**						−0.12†		−0.26*			−0.39***
Grey silverfish	−0.17*	0.16*	−0.17†						−0.21†	0.22†		−0.33***
Bed bug	−0.11†											−0.18**
Cockroach	−0.16*					0.15†						−0.22***
Furniture beetle	−0.17*											−0.24***
Biscuit beetle	−0.14*					0.18*						−0.32***
Ladybird beetle	−0.14*						−0.12†					−0.32***
**IA avoidance**												
Housefly	−0.15*											
Grey silverfish	−0.18*											−0.21**
Bed bug												−0.12†
Cockroach												
Furniture beetle												
Biscuit beetle												−0.12†
Ladybird beetle												

Significant (*P < 0.05; **P < 0.01; ***P < 0.001) and marginally significant (†P < 0.1) standardised regression coefficients (beta) are presented. Blank spaces represent non‐significant betas.

**Table 11:** The relationships between information‐seeking behaviour (ISB), information avoidance (IA) measured as the extent to which seeking information about the subject is a waste of time (waste of time) and information avoidance (IA) measured as the extent to which information about the subject is avoided (avoidance) and the dependent variables behavioural intention (BI) measured as the extent to which a person takes preventive measures (preventive) and BI measured as the extent to which curative measures will be taken (curative) for different insects

	Predictors		
		IA	
Dependent	ISB	Waste of time	Avoidance
**BI preventive**			
Housefly	0.25***		−0.14*
Grey silverfish	0.26***	−0.21**	
Bed bug	0.27***		
Cockroach	0.24**		
Furniture beetle	0.15†		
Biscuit beetle	0.20**		
Ladybird beetle	0.42***		
**BI curative**			
Housefly	0.40***		
Grey silverfish	0.73***	−0.21**	
Bed bug	0.66***		
Cockroach	0.39***	−0.21**	
Furniture beetle	0.47***		
Biscuit beetle	0.55***		
Ladybird beetle	0.47***		

Significant (*P < 0.05; **P < 0.01; ***P < 0.001) and marginally significant (†P < 0.1) standardised regression coefficients (beta) are presented. Blank spaces represent non‐significant betas.

**Table 12:** The relationships between tolerance level (TL) measured as the extent to which the presence of insects is tolerated (tolerable) and TL measured as the extent to which a person is prepared to pay for control measures (will pay) and the dependent variables behavioural intention (BI) measured as the extent to which a person takes preventive measures (preventive) and BI measured as the extent to which curative measures will be taken (curative) for different insects

	Predictors	
	TL	
Dependent	Tolerable	Will pay
**BI preventive**		
Housefly	−0.10†	0.31***
Grey silverfish		0.35***
Bed bug		
Cockroach		0.23***
Furniture beetle	0.14*	0.12†
Biscuit beetle		0.20**
Ladybird beetle		0.43***
**BI curative**		
Housefly	−0.29***	0.27***
Grey silverfish	−0.19***	0.43***
Bed bug	−0.11*	0.48***
Cockroach		0.54***
Furniture beetle		0.50***
Biscuit beetle	−0.13*	0.43***
Ladybird beetle	−0.21**	0.21***

Significant (*P < 0.05; **P < 0.01; ***P < 0.001) and marginally significant (†P < 0.1) standardised regression coefficients (beta) are presented. Blank spaces represent non‐significant betas.

### 3.1 Perceived hazard characteristics (risk)

In general, age and RHE were related to PHC (risk). Both showed a significant positive relationship for four insects (age: housefly, bed bug, cockroach and biscuit beetle; RHE: housefly, grey silverfish, furniture beetle and ladybird beetle), and age showed a marginally significant positive relationship for the ladybird beetle and RHE a marginally significant positive relationship for the bed bug (Table 6). Mean values and standard deviation of RHE for the biscuit beetle and cockroach were very low, which may explain why a relationship could not be found (Table 4). A relationship between age and PHC (risk) was not found for the ladybird beetle or furniture beetle. The perceived risks of the furniture beetle, cockroach, and bed bug were highest, whereas the perceived risks of the ladybird beetle were low (Table 4).

### 3.2 Informational subjective norms

Age and having pets were the only demographic factors that showed a relationship with ISN. A positive significant relationship existed for the housefly for age and having pets, and for the grey silverfish for age. A positively marginally significant relationship was shown for age for the bed bug. Furthermore, a highly significant positive relationship was found between RHE and ISN for the housefly, grey silverfish and ladybird beetle (Table 6). Mean values of RHE were significantly higher for these insects than for the other insects tested (Table 4).

### 3.3 Affective responses

No relationships were found between PHC (attributes) and PHC (risk) and AR (pos) for a majority of the insects tested. PHC (risk) was, however, strongly and positively associated with AR (neg) for all insects (Table 7). Of the various predictors of PHC (attributes), only insects looking scary showed a positive significant relationship with AR (neg) for the majority of insects (bed bug, cockroach, furniture beetle and biscuit beetle).

Mean values for AR (pos) were low for all insects, with the ladybird beetle scoring significantly higher than all other insects. Mean values of AR (neg) were higher than values of AR (pos), with highest values for the bed bug and cockroach and lowest for the ladybird beetle (Table 4).

### 3.4 Tolerance levels

For nearly all insects tested, looking scary and disgusting showed significant relationships with TL (tolerable). The ladybird beetle (marginally significant for looking scary) and housefly (no significant relationship for both predictors) were the only exceptions (Table 8). The insects looking scary showed a positive relationship, whereas looking disgusting showed a negative relationship with TL (tolerable) for the insects, except for the ladybird beetle, which expressed an opposite relationship for both. A significant negative relationship was shown for flying and TL (tolerable) for all insects except the ladybird beetle, whereas there was a (marginally) significant positive relationship between crawling or jumping and TL (tolerable) for all insects except the ladybird beetle. PHC (risk) and both positive and negative ARs did not show a significant relationship with TL (tolerable) for the majority of insects.

In contrast, both PHC (risk) and AR (neg) showed a highly significant positive relationship with TL (will pay), with the exception of PHC (risk) for the housefly (significant) and the ladybird beetle (marginally significant). Of the predictors from PHC (attributes), only looking disgusting showed a significant positive relationship for the majority of insects (Table 8).

Mean values of TL (tolerable) were low for the bed bug, cockroach, furniture beetle and biscuit beetle; these values were significantly lower than the mean value for the grey silverfish, which was significantly lower than the mean value for the housefly. The ladybird beetle scored the highest mean value, being significantly higher than those of all other insects.

Mean values of TL (will pay) were high for the bed bug, cockroach and furniture beetle. They were significantly higher than those for the biscuit beetle and grey silverfish, which were significantly higher than the mean value for the housefly. The mean value for the ladybird beetle was significantly lower than those for all other species (Table 4).

### 3.5 Information sufficiency

Only TL (will pay) showed a (positive) relationship with IS for all insects. TL (tolerable) and ISN did not show a significant relationship with IS for the majority of insects (Table 9).

IS was positive and highest for the biscuit beetle, bed bug, furniture beetle and cockroach (in descending order). Only 15.6% of the respondents reported knowing the biscuit beetle, whereas 38.4%, 69.6%, and 79.8% were familiar with the bed bug, furniture beetle and cockroach, respectively. The housefly and ladybird beetle were known by nearly all respondents (98.5% and 99.6%, respectively), and showed a negative mean IS value, meaning that respondents possessed sufficient information to be able to cope with the risks associated with these insects (Table 4).

### 3.6 Information‐seeking behaviour

The various predictors were not consistently significantly related with IA (avoidance) for the different insects. There was, however, a highly significant relationship between IS and ISB for all insects. Furthermore, there was a (marginally) significant relationship between the relevant belief in online forums and ISB (Table 10). RCB for online forums was comparable to family, websites of scientific institutions and local governments, and books. The mean value of RCB for online forums was lower than those for the PCO websites, PCOs and insecticide labels or salespersons, but higher than those for local government employees, neighbours, newspapers or television, and employees of scientific institutions (Table 5).

A highly significant negative relationship for all insects was found between IS and IA (waste of time), as well as a significant negative relationship between PIGC and IA (waste of time) for all insects except bed bugs and furniture beetles (both marginally significant). Respondents had a mean value of 5.1 for PIGC (Table 5).

### 3.7 Behavioural intention

ISB was highly significantly related to both BI (preventive) and BI (curative) for all insects, except for BI for the furniture beetle (preventive; marginally significant). IA (waste of time) and IA (avoidance) did not show a significant relationship with BI (preventive) and BI (curative) for the majority of insects (Table 11). Five of seven insects (all except the bed bug and furniture beetle, which were not and moderately significant, respectively) showed a significant relationship between TL (will pay) and BI (preventive). A significant relationship between TL (tolerable) and BI (preventive) was found for the furniture beetle (Table 12).

TL (will pay) was highly significantly positively related to BI (curative) for all insects, whereas TL (tolerable) was significantly negatively related to BI (curative) for five insects; no significant relationship was found for the furniture beetle and cockroach.

In general, mean values of BI (curative) were higher than those of BI (preventive). BI (preventive) was highest for the housefly and cockroach, and lowest for the ladybird beetle. The cockroach, furniture beetle and bed bug showed the highest values and the ladybird beetle showed the lowest values for BI (curative) (Table 4).

### 3.8 Overview of general model

**Figure 2:**
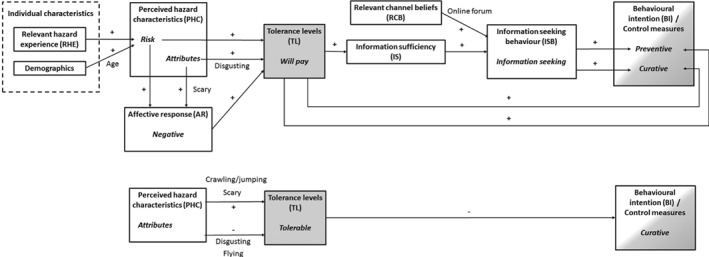
Results of the generalised integrated model showing significant relationships (+, positive relationship; –, negative relationship).

The general theoretical model that was created by testing the relationships of variables from the RISP model and IPM framework is shown for the two items of TL in Figure 2. For the generalisation of the model, relationships were consolidated into the model when over half of the insects showed a significant relationship. For reasons of parsimony, relationships leading to a dead end have been removed [i.e. the negative relationship between the predictor variables IS and PIGC and the dependent variable IA (avoidance)].

## 4 DISCUSSION

By combining variables from the RISP model and IPM framework, an integrative model was developed in which a sequence of relationships between personal factors and TL, and between TL, ISB and intentional pest management behaviour in the household was examined. Understanding the relationships between these factors can improve information provision to the public, which is an important step in the process of IPM.^9^

For the sake of the discussion, the general model is divided into two sections: (1) the relationships between personal factors and TL and (2) the relationships between TL, information seeking, and BI. Furthermore, practical implications and study limitations are discussed.

### 4.1 Relationships between personal factors and tolerance levels

In line with other studies, tolerance for the selected insects in our study was low.^8^, ^11^, ^35^, ^36^ As expected, there was variation in tolerance between the different insect species, with the ladybird beetle being tolerated the most and cockroaches and bed bugs the least.

In addition to the perceived risk (a product of the severity of the consequences of the insects' presence and the perceived chance of encountering the insects indoors), the mobile and cosmetic or aesthetic characteristics of insects, i.e. flying, jumping or crawling, or looking disgusting and/or scary, especially affected tolerance for the various insects. As in our study, in a previous study, aesthetic qualities such as being ugly, nasty, scary or disturbing resulted in the selection of cockroaches as the least‐liked animals.^37^ In fact, only these aspects affected the extent to which the presence of insects was tolerable. A relationship between the risk of damaging health, materials, construction or food and tolerance levels was not found in our study, although these factors were considered of great concern to the respondents. This is in accordance with other studies where it was found that intolerance of insects in the household environment existed regardless of the risk they pose to health or property.^8^, ^36^

Both the perceived risk and negative AR only showed a positive relationship with the willingness to pay for control. It is not surprising that positive feelings scored lower than negative feelings. In an earlier study, it was found that only 13% of respondents liked insects they encountered within their homes, and 86% disliked or were afraid of them.^35^ Risk perception affected the negative AR, whereas risk perception was affected by individual factors, according to the RISP model.

In this study, both RHE and age affected the perceived risk, with a positive relationship for the majority of insects tested. Other studies have shown a relationship between other demographic factors and attitudes^36^ or tolerance.^8^, ^35^ If, however, the insects in our study were regarded separately, other demographic factors, such as gender, level of education and having children or pets, were also found to have an influence. The relationship between RHE and perceived risk was not found for the biscuit beetle, bed bug or cockroach, possibly because of the lack of experience with these insects, resulting in a lack of variation in data.

In general, RHE and age affected the risk perception, which in turn showed a positive relationship with the adverse feelings people had about a specific insect. Both the perceived risk and perception of aesthetic attributes of insects, together with the negative AR, drove the willingness to pay for extermination, whereas only the perception of mobility and aesthetic attributes of insects determined whether the insects were tolerable within the household environment.

### 4.2 Relationships between tolerance levels, information‐seeking behaviour and behavioural intentions

The difference between the two aspects of TL (willingness to pay for extermination and the extent to which the insects are tolerable) was found in the second, behavioural, part of the model as well (Figure 2). The extent to which the presence of insects was tolerable did not fit the theory of the RISP model. It was not a predictor for IS and, subsequently, ISB. It was, however, negatively related to curative (extermination or removal of insects present) BI for the majority of insects tested. The extent to which an insect's presence was tolerated was based on mobile and aesthetic characteristics of the insect, and predicted curative control behaviour only. It reflects the less sustainable ‘quick fix’ approach to pest control, in which non‐tolerated insects are simply exterminated without obtaining information about the species or taking possible preventive measures into account.^19^

This is in contrast to the TL expressed in monetary terms (willingness to pay for extermination). The latter showed a stronger accordance with the RISP model and findings of other studies. Risk judgement and AR were, for instance, consistently related to attitudes and BI of cancer patients enrolling in clinical trials.^38^ The willingness to pay for control affected the need for information (or IS), which, in turn, together with the RCB for online fora, affected ISB. Furthermore, the willingness to pay and ISB affected both preventive and curative BI. People were not only interested in getting rid of the insect pest, but were willing to take measures to prevent future infestations as well. This is in agreement with one of the fundamental principles of IPM: understanding why pests are present and eliminating the cause of their presence.^9^ It can therefore be concluded that only TL measured as the extent to which a person is willing to pay for control measures provides a valid integrated model of RISP and IPM.

If tolerance for insects in the household environment is lower (individuals are more willing to pay for extermination), the need for information to cope with the risks associated with the presence of insects is higher. In our study, IS was negative for the housefly and ladybird beetle only. These were the only insect species tested that people were confident about having the information and knowledge to cope with in terms of risk. This may be because of the relatively low risk perception and negative AR for both species. More information was needed to be able to handle the risks that other insects posed, with the lowest IS for the biscuit beetle, with which only 16% of the respondents were familiar.

When searching for information, the sources having practical control associations (PCO websites, PCOs and insecticide labels or salespersons) scored highest on RCB. In turn, websites and books scored higher than employees of local governments and scientific institutions, neighbours and newspapers or television. It may be harder to consult with a person from the local government or scientific institution about pest problems than PCOs or an insecticide label. The product label indeed is an important source of information for consumers, even though the label is often not completely understood or is found to be incomplete by the user.^15^ Of all sources, only the RCB for online fora was positively related to ISB. Online fora enable people to take advantage of past experiences from multiple peers who have encountered similar challenges. Furthermore, the information provided by these peers may be perceived as more neutral than the information provided by PCOs, the local government, newspapers and other sources.

As expected, based on the TL and IS, the cockroach, bed bug, and furniture beetle scored high on ISB, whereas the only species with negative IS, the housefly and ladybird beetle, scored lowest. IS does not, however, measure the actual knowledge an individual possesses, and it would be interesting to study the actual knowledge about these species and their management. It is possible that persons with a negative IS for an encountered insect use an inadequate or unsustainable pest management approach, because they possess inadequate knowledge but at the same time do not search for information for correct management.

Considering BI, people were more willing to take curative (e.g. insecticidal sprays, cleaning or removal) than preventive measures. This may not be surprising, as curative measures mitigate a risk that is actually present, whereas preventive measures require individuals to perform activities and spend money to lower a potential risk associated with the hypothetical chance of future insect pest presence.^39^ The intention to take curative or preventive measures was lowest for the ladybird beetle, the insect that was regarded to be the least risky, elicited the fewest negative feelings and was tolerated most of all tested insects. People were most willing to take preventive measures for the housefly and curative measures for the bed bug, cockroach and furniture beetle. The positive relationship between both the willingness to pay for extermination and ISB and the BI of preventive measures suggests that especially the individuals with a low TL (will pay) can be persuaded to take preventive measures.

### 4.3 Practical implications

In general, important factors that drove TL were age, experience, risk perception and negative AR. Altering a person's tolerance for a certain insect is most likely to be achieved by shifting its risk perception, as age and experience cannot really be altered and risk perception drives negative emotions as well. Both risk perception and negative emotions are negatively related to tolerance. As TLs drive the need for information, and the need for information and confidence in online fora in turn drive ISB, providing pest management information to a person with a low risk perception may not be effective. People who are not interested in information about the prevention and control of insect pests in the household environment because of low risk perception may not process information about insect management, such as the importance of hygienic measures, and caulking cracks and crevices for effective cockroach control, and may be difficult to convince to use preventive or curative pest management tactics.^35^, ^40^ Providing individuals with the information to assess the perceived risk of the cockroaches realistically, instead of directly providing information about cockroach management, may be more effective to eventually alter their control behaviour. Focusing on risk‐related information has been suggested to increase ISB about clinical trial enrolment as well.^44^

Although this strategy can be applied generally, the differences in relevant driving factors that have been found between the tested insects have implications for communication strategies. For instance, in addition to age, having children had a positive relationship with risk perception of bed bugs. In the case of bed bugs, it is expected that the elderly and families with children process provided bed bug management information. For other demographic groups, however, information regarding the risks of bed bug infestations must be provided as well. As information is often provided to communities as part of IPM strategies in multi‐housing units or neighbourhoods,^19^, ^40^, ^42^, ^43^ providing risk‐related information before an informational meeting may increase attendance.

Furthermore, understanding the confidence in sources of information is important to be able to provide information effectively. Our results suggest that an online forum about pest management may increase ISB for all insects tested. Because of the wide range of information provided by the public on forums, this may be a risk and it should at least be moderated by a pest management expert. The RCB for other sources drove ISB as well. RCB for websites of PCOs drove ISB for bed bugs, cockroaches and furniture beetles, whereas this was the case for family with the housefly. Using family as a source of information may pose a risk, as this may not be reliable, and this should be taken into account when dealing with housefly pest situations.

Because of the variation in relevant factors between insects, especially demographics and RCB for different sources, performing a survey of a specific insect species will provide valuable information about how information is best provided to target audiences.

### 4.4 Limitations of the study and future research

It should be noted that the proposed model is based on general relationships that were found for at least the majority of insects tested. Because of the design, in which seven insects were used, the questionnaire may have seemed repetitive. By including an insect that was expected to elicit opposite responses (ladybird beetle) to the pest insects, it was shown that respondents were conscientious in answering the questions until the end of the survey, as the descriptive results for this insect were consistently significantly different from those for most other species throughout the survey.

Furthermore, we did not test all relationships of the RISP model, such as the relationship between personal factors and RCB, because they were not within the focus of our study. They may, however, be of interest in practical situations.

Finally, this study focused on the risks associated with insects, whereas the risks of control measures were not considered. As risks are associated with certain control tactics (such as insecticide use), a similar model could be developed for different management tactics, both with and without chemicals. It would be interesting to identify whether there is a relationship between the BI, tolerance for insects and tolerance for management tactics, such as chemical as well as non‐chemical and novel insecticide use.^44^, ^45^ It is hypothesised that tactics that are considered more risky are applied only if the TL of a certain tactic exceeds the TL of a specific insect. Understanding this relationship may further improve the communication aspects of IPM strategies.

## 5 CONCLUSIONS

The aim of this study was to test whether the proposed model is valid, what factors are most important and whether this model is general or insect specific. We demonstrated that it is possible to integrate the RISP model within IPM. The measurement of TL is of importance, as a sequence of relationships between personal factors, TL, ISB and BI was found when TL was measured as the extent to which people are willing to pay for pest control, but not when it was measured as the extent to which a person believes the presence of household insects is tolerable.

In general, the relevant driving factors that determine a person's TL in this model were age, experience, risk perception, and negative AR. TL, RCB for online forums, IS and ISB were relevant factors driving BI for pest management, but differences in relevant factors were found between insects.

## Appendix 1 1 Survey questions

Translations of questions of the survey are shown, with the respective variable from the model. The majority of responses were given according to a seven‐point Likert scale, with 1 = not at all and 7 = to a large extent.

**Table:**

Variable	Question	Response
	Do you know these insects? Housefly, grey silverfish, bed bug, ladybird beetle, biscuit beetle, furniture beetle, cockroach	Yes/no
RHE	Have you ever encountered these insects at home? Housefly, grey silverfish, bed bug, ladybird beetle, biscuit beetle, furniture beetle, cockroach	Seven‐point Likert scale
	Have you ever experienced nuisance from these insects at home? Housefly, grey silverfish, bed bug, ladybird beetle, biscuit beetle, furniture beetle, cockroach	Seven‐point Likert scale
AR	When thinking of the presence of houseflies at home I feel: Anxious, ashamed, comfortable, content, enthusiastic, uncomfortable, worried	Seven‐point Likert scale
	When thinking of the presence of grey silverfish at home I feel: Anxious, ashamed, comfortable, content, enthusiastic, uncomfortable, worried	Seven‐point Likert scale
	When thinking of the presence of bed bugs at home I feel: Anxious, ashamed, comfortable, content, enthusiastic, uncomfortable, worried	Seven‐point Likert scale
	When thinking of the presence of ladybird beetles at home I feel: Anxious, ashamed, comfortable, content, enthusiastic, uncomfortable, worried	Seven‐point Likert scale
	When thinking of the presence of biscuit beetles at home I feel: Anxious, ashamed, comfortable, content, enthusiastic, uncomfortable, worried	Seven‐point Likert scale
	When thinking of the presence of furniture beetles at home I feel: Anxious, ashamed, comfortable, content, enthusiastic, uncomfortable, worried	Seven‐point Likert scale
	When thinking of the presence of cockroaches at home I feel: Anxious, ashamed, comfortable, content, enthusiastic, uncomfortable, worried	Seven‐point Likert scale
PHC	To what extent do you consider the consequences caused by these insects at home as a nuisance? Housefly, grey silverfish, bed bug, ladybird beetle, biscuit beetle, furniture beetle, cockroach	Seven‐point Likert scale
	To what extent do you think that you will suffer the consequences of these insects at home? Housefly, grey silverfish, bed bug, ladybird beetle, biscuit beetle, furniture beetle, cockroach	Seven‐point Likert scale
	To what extent do you consider these insect attributes a nuisance? Damage food, damage materials, damage construction, stinging or biting, vector of disease‐causing pathogens, being plentiful, looking scary, looking disgusting, flying, crawling or jumping, indoor reproduction, outdoor reproduction, and difficult to control	Seven‐point Likert scale
ISN	To what extent do residents expect you to search for information regarding these insects at home? Housefly, grey silverfish, bed bug, ladybird beetle, biscuit beetle, furniture beetle, cockroach	Seven‐point Likert scale
	To what extent do people you find important expect you to have knowledge about these insects at home? Housefly, grey silverfish, bed bug, ladybird beetle, biscuit beetle, furniture beetle, cockroach	Seven‐point Likert scale
TL	To what extent do you consider the presence of these insects at home tolerable? Housefly, grey silverfish, bed bug, ladybird beetle, biscuit beetle, furniture beetle, cockroach	Seven‐point Likert scale
	To what extent are you willing to pay to get rid of these insects at home? Housefly, grey silverfish, bed bug, ladybird beetle, biscuit beetle, furniture beetle, cockroach	Seven‐point Likert scale
IS	I know what nuisance these insects may cause and possess sufficient information to prevent this. Housefly, grey silverfish, bed bug, ladybird beetle, biscuit beetle, furniture beetle, cockroach	Seven‐point Likert scale
	I know enough about these insects to be able to control them successfully. Housefly, grey silverfish, bed bug, ladybird beetle, biscuit beetle, furniture beetle, cockroach	Seven‐point Likert scale
	I need more information to prevent nuisance from these insects. Housefly, grey silverfish, bed bug, ladybird beetle, biscuit beetle, furniture beetle, cockroach	Seven‐point Likert scale
	I need much more information to be able to prevent nuisance from these insects. Housefly, grey silverfish, bed bug, ladybird beetle, biscuit beetle, furniture beetle, cockroach	Seven‐point Likert scale
	I need much more information to be able to control these insects. Housefly, grey silverfish, bed bug, ladybird beetle, biscuit beetle, furniture beetle, cockroach	Seven‐point Likert scale
PIGC	To what extent do the following statements apply to you? I know where to find information regarding insects at home. I can easily find time to collect information regarding insects at home.	Seven‐point Likert scale
RCB	To what extent would you use these sources when searching for information regarding insects at home? Family, neighbours, pest control operators (PCOs), employees of local government, employees of scientific universities, museums, and centres of expertise, insecticide label or salespersons, books, newspapers or television, PCO websites, local government websites, scientific institution websites, and online forums	Seven‐point Likert scale
	To what extent do you consider these sources useful? Family, neighbours, pest control operators (PCOs), employees of local government, employees of scientific universities, museums, and centres of expertise, insecticide label or salespersons, books, newspapers or television, PCO websites, local government websites, scientific institution websites, and online forums	Seven‐point Likert scale
	To what extent do you consider these sources accessible? Family, neighbours, pest control operators (PCOs), employees of local government, employees of scientific universities, museums, and centres of expertise, insecticide label or salespersons, books, newspapers or television, PCO websites, local government websites, scientific institution websites, and online forums	Seven‐point Likert scale
	To what extent do you consider these sources reliable? Family, neighbours, pest control operators (PCOs), employees of local government, employees of scientific universities, museums, and centres of expertise, insecticide label or salespersons, books, newspapers or television, PCO websites, local government websites, scientific institution websites, and online forums	Seven‐point Likert scale
ISB	If I encountered these insects at home I would search for information about them.	Seven‐point Likert scale
	Housefly, grey silverfish, bed bug, ladybird beetle, biscuit beetle, furniture beetle, cockroach	
	If these insects caused a nuisance at home, I would search for information about them. Housefly, grey silverfish, bed bug, ladybird beetle, biscuit beetle, furniture beetle, cockroach	Seven‐point Likert scale
	Information‐seeking regarding these insects is a waste of time. Housefly, grey silverfish, bed bug, ladybird beetle, biscuit beetle, furniture beetle, cockroach	Seven‐point Likert scale
	I avoid information about these insects and do not want to know anything about them. Housefly, grey silverfish, bed bug, ladybird beetle, biscuit beetle, furniture beetle, cockroach	Seven‐point Likert scale
BI	I take measures to prevent nuisance from these insects. Housefly, grey silverfish, bed bug, ladybird beetle, biscuit beetle, furniture beetle, cockroach	Seven‐point Likert scale
	When experiencing nuisance I will exterminate or remove these insects. Housefly, grey silverfish, bed bug, ladybird beetle, biscuit beetle, furniture beetle, cockroach	Seven‐point Likert scale
Demographics	Gender?	Male/female
	Age?	Open
	Residence?	Open
	Highest education?	Multiple choice
	Property owned or rented?	Owned/rented
	Children (<18) at home?	Yes/no
	Pets?	Yes/no
	Involved with insects for profession or hobby?	Yes/no
